# Recent Advances in Polypeptide Antibiotics Derived from Marine Microorganisms

**DOI:** 10.3390/md21100547

**Published:** 2023-10-22

**Authors:** Wei Wang, Liting Gu, Jiahao Wang, Xubin Hu, Bin Wei, Huawei Zhang, Hong Wang, Jianwei Chen

**Affiliations:** Key Laboratory for Green Pharmaceutical Technologies and Related Equipment of Ministry of Education & Key Laboratory Pharmaceutical Engineering of Zhejiang Province & College of Pharmaceutical Science & Collaborative Innovation Center of Yangtze River Delta Region Green Pharmaceuticals, Zhejiang University of Technology, Hangzhou 310014, China

**Keywords:** antimicrobial activity, depsipeptides, lipopeptides, oligopeptides, antibiotic resistance

## Abstract

In the post-antibiotic era, the rapid development of antibiotic resistance and the shortage of available antibiotics are triggering a new health-care crisis. The discovery of novel and potent antibiotics to extend the antibiotic pipeline is urgent. Small-molecule antimicrobial peptides have a wide variety of antimicrobial spectra and multiple innovative antimicrobial mechanisms due to their rich structural diversity. Consequently, they have become a new research hotspot and are considered to be promising candidates for next-generation antibiotics. Therefore, we have compiled a collection of small-molecule antimicrobial peptides derived from marine microorganisms from the last fifteen years to show the recent advances in this field. We categorize these compounds into three classes—cyclic oligopeptides, cyclic depsipeptides, and cyclic lipopeptides—according to their structural features, and present their sources, structures, and antimicrobial spectrums, with a discussion of the structure activity relationships and mechanisms of action of some compounds.

## 1. Introduction

The discovery of antibiotics dramatically reduced the threat of death when people experienced wound infections and made surgery a widely acceptable option for treatment [1,2]. However, the presence of antibiotic resistance persists, making the risk of death from bacterial infections ever present and getting worse with the rapid development of superbugs [3,4,5]. A recent research study suggests that bacterial infections are associated with 13.6% of global deaths in 2019 and have become the second leading cause of death worldwide after ischemic heart disease [6]. More worryingly, like the ongoing global spread of mobile colistin resistance gene (MCR-1)-mediated resistance against carbapenems and colistin, the development of super-resistant bacteria is exacerbating the threat to global medical practices [7,8,9,10]. Many recent studies are showing that resistance to superbugs continues to develop and increase, such as methicillin-resistant *Staphylococcus aureus* (MRSA), and that the number of super-resistant bacterial species is increasing, such as the epidemics in recent years of carbapenem-resistant *Klebsiella pneumoniae* and carbapenem-resistant *Pseudomonas aeruginosa* [11,12,13,14,15]. The prevalence of these superbugs has made treatment more difficult for many patients, such as cancer patients who have to face a significant threat of death from bacterial infections while undergoing chemotherapy or surgery [16]. At the same time, the number of both clinically available and in-development antibiotics is rapidly decreasing [17,18,19].

To cope with the increasing antibiotic resistance crisis, the extension of backup antibiotic pipelines is urgent. Antimicrobial agents come from a variety of sources, such as microbes, insects, plants, fishes, mollusks, etc. [20,21,22,23,24]. Recently, the discovery of cyclic peptide antibiotics derived from microorganisms has become a new research hotspot, attracting the attention of many researchers. The structural diversity of peptide antibiotics generates a diverse and broad antimicrobial spectrum. For example, cilagicin [25], macolacin [26], and lugdunin [27,28,29], which are effective in killing Gram-positive bacteria, as well as darobactin [30,31] and dynobactin A [32], which attack Gram-negative bacteria. Meanwhile, cyclic peptide antibiotics also have various antibacterial mechanisms, such as, corbomycin can inhibit peptidoglycan remodeling [33], evybactin acts on DNA gyrase [34], klebsazolicin targets ribosomes [35], colistin binds to lipid A in the cell membrane [36], teixobactin inhibits the synthesis of the cell wall [37], and so forth. Considering the remarkable potential of cyclic peptide antibiotics, we have compiled recent research advances on cyclic peptide antibiotics derived from marine microorganisms and introduced their structures, sources, antimicrobial spectra, as well as some of the explored antimicrobial mechanisms and brief structure activity relationships in this review. The microbial origins of the compounds we have discussed were clearly identified to avoid, as far as possible, the misattribution of compounds to macro-organisms to which the actual microbial producers belong or are associated. It is worth noting that the peptide antibiotics we have compiled distinguish themselves from antimicrobial peptides, also called host defense peptides [38]. Antimicrobial peptides tend to have molecular weights above 2000 Da, two-dimensional structures such as α-helix or β-folding or even more complex three-dimensional structures, and physicochemical properties more similar to proteins [39,40,41,42]. The peptides we organize are around or below 1000 Da, do not have a two-dimensional structure, and are more similar to small molecule compounds. In addition, some of the compounds we have compiled contain antifungal activity, such as sclerotides, iturins, hassallidins and balticidins. Although fungal infections are much less of a threat than superbugs, the discovery of new antifungal agents could further enrich the clinical therapeutic options.

## 2. Cyclic Oligopeptides

Cyclic oligopeptides are cyclic peptides consisting of 2–20 amino acids that are mostly synthesized by non-ribosomal peptide synthetase. The amino acids forming the cyclic skeleton are all linked by amide bonds [43]. Some group modifications may be present on the side chain of amino acid residues or on the N atom of the amide bond. Linear branches may extend from the cyclic skeleton when lysine (Lys), ornithine (Orn), or other amino acid residues with free amino groups on the side chain are present to participate in cyclization.

### 2.1. Ilamycins

Ilamycins are cyclic heptapeptides with the same type of skeleton. This family has a large number of members, such as ilamycin B1–F (1–8, Table 1) from *Streptomyces atratus* SCSIO ZH16, and ilamycins G–R (9–20, Table 1) from artificial mutant strain *Streptomyces atratus* SCSIO ZH16 ΔilaR [44,45,46]. Four of seven amino acid residues in ilamycins are highly conserved, including alanine (Ala), two leucine (Leu) residues, and one L-2-amino-4-hexenoic acid. The chemical structural diversity arises from the nitro modification of the adjacent hydroxyl position on tyrosine (Tyr), the modified portion on the imino of Try, and the presence of multiple post-modified Leu side chains. Ilamycins are not active against Gram-positive or Gram-negative bacteria in general, but they have impactful anti-*M. tuberculosis* activity, with ilamycin J (12, Table 1) reaching a minimal inhibitory concentration (MIC) of 9.6 nM against *M. tuberculosis* HR37v and ilamycins E1/E2 (6/7, Table 1) having an MIC of 9.8 nM [44,45]. An SAR investigation between analogues showed that Leu with modified side chains has an important effect on the anti-TB activity, which is enhanced when the side chain is synthesized into a hexameric ring with an adjacent imino and diminished when the side chain is oxidized to form hydroxyl groups [47]. The activity of the modified portion on tryptophane (Trp) is weakened or not affected by oxidation to form hydroxyl groups, but is greatly enhanced when the alkenyl group is oxidized to an epoxy group. In addition, the nitro group on Tyr also has an important effect on the activity, as the group’s presence significantly enhances it [48]. The total synthesis of ilamycins E1 (6, Table 1) and F (8, Table 1) has been accomplished, which will make it possible to explore more comprehensive and detailed structure–activity relationships (SARs) [48,49].

Tyrosine hydrolytic protein C1 (ClpC1) is an adenosine triphosphate (ATP)-dependent homologue of the ClpC family of concomitant proteins in *M. tuberculosis* that acts as the site of action for ilamycins [45,46]. ClpC1 binds to the protein hydrolysis structural domains ClpP1 and ClpP2 and is jointly responsible for the degradation of intracellular waste proteins in *M. tuberculosis* [50]. Because ClpC1 is required for efficient protein degradation using ATP, these protein hydrolysis structural domains can only perform low levels of unregulated protein degradation in the absence of ClpC1. Thus, ClpP protease-mediated protein hydrolysis in *M. tuberculosis* is tightly regulated by ClpC1 [50]. The ClpC1 protein has three structural domains, including a N-terminal helix domain (NTD) and nucleotide-binding structural domains D1 and D2 [44]. Ilamycins can bind to ClpC1-NTD and inhibit the hydrolytic activity of the ClpC1/ClpP1/ClpP2 protein complex. This is in contrast to the known mechanism of action of cyclomarin A [51] or lassomycin [52], which also acts on ClpC1 by stimulating ATPase, promoting the hydrolytic activity of the ClpC1/ClpP1/ClpP2 protein complex or uncoupling ClpC1 from ClpP1/ClpP2 [53,54].

### 2.2. Asperversiamides and Asperheptatides

Asperversiamides A–C (21–23, Table 1) are cyclic heptapeptides isolated from a coral-derived fungal strain *Aspergillus versicolor* CHNSCLM-0063 with strong anti-*Mycobacterium marinum* activity, where asperversiamide B also has mild anti-TB activity [55]. asperheptatides A–D (24–27) are derived from the same fungus, share the same cyclic heptapeptide skeleton as asperversiamides, and have the following amino acid residue sequence: cyclo-Trp1-D-Ala/serine z(Ser)2-Ala/glycine(Gly)/phenylalanine(Phe)3-D/L-valine(Val)4-D-Phe5-Val6-Ala/Ser7 (Table 1) [56,57]. Asperheptatides A–B are structurally closest to asperversiamide B and also exhibit mild anti-TB activity [56]. Because cinnamic acid is an important structure in anti-TB antibiotics, it might be possible to obtain derivatives with greater activity via cinnamic acid modification [58]. Using asperversiamide A as a skeleton, various cinnamic acid groups have been introduced on the hydroxyl group of the serine side chain, obtaining cinnamic acid derivatives (28–31, Table 1) with an eight-fold increase in anti-TB activity [56]. This also demonstrated the anti-TB effect of the cinnamic acid structure.

### 2.3. Thermoactinoamides

Thermoactinoamides, isolated from the thermophilic bacterium *Thermoactinomyces vulgaris* ISCAR 2354, are cyclic hexapeptides containing five common lipophilic amino acid residues and one aromatic amino acid residue [59]. The amino acid sequence of thermoactinoamide A (32, Table 1) is cyclo-D-Tyr1-Val2-Leu3-D-Leu4-Leu5-D-Isoleucine (Ile)6. Except for thermoactinoamide A, B–F are not isolated to the compound monomer, and the structure of B–F is postulated on the basis of the skeleton for A and mass spectrometry analysis. Thermoactinoamide A has a narrow antimicrobial spectrum, exhibiting selective growth inhibition only against *S. aureus* (MIC, 35 μM) [59]. Because antibacterial data were obtained only for thermoactinoamide A, the antibacterial ability of thermoactinoamide skeleton still needs to be explored.

### 2.4. Desotamides A–D

Desotamides A–D and G (33–37, Table 1), isolated from *Streptomyces scopuliridis* SCSIO ZJ46, are cyclic hexapeptides that inhibit the growth of Gram-positive bacteria [60,61,62]. The amino acid sequence of desotamide B (34, Table 1) is cyclo-D-Leu1-Leu2-Trp3-Gly4-Asparagine (Asn)5-allo-Ile6, which was further confirmed by total synthesis [61,63]. The unique N-formyl-kynurenine residue replaces Trp3 in desotamide C (35, Table 1) and kynurenine in desotamide D (36, Table 1) [61]. Desotamide B demonstrate inhibitory effects on *S. aureus*, *S. pneumiae*, and methicillin-resistant *Staphylococcus epidermidis* (MRSE) with MICs of 16.0, 12.5, and 32.0 μg/mL, respectively. On the other hand, desotamides C–D do not exhibit any activity, suggesting an important role for Trp in the skeleton [61]. Further SAR investigation has shown that the anti-Mycobacterium activity decreases when D-Orn is replaced by D-Lys or D-Arg and is lost when replaced by L-Lys or L-Arg (Arg) [64]. Therefore, Leu2 and Trp3 are once again shown to be indispensable, while amino acid residues at other positions allow for appropriate modifications [64].

### 2.5. Surugamides

Surugamides A–E (38–42, Table 1) are cyclic octapeptides isolated from *Streptomyces* sp. JAMM992 from deep-sea sediment and are rich in hydrophobic amino acid residues [65]. Another study showed that surugamide A (38, Table 1) is also available from the marine sponge isolate *Streptomyces sp.* SM17 [66]. The amino acid residue sequence for surugamide A is Ile1-D-allo-Ile2-Lys3-Ile4-D-Phe5-D-allo-Leu6-Ile7-D-Ala8, of which four Ile are substituted by Val in sequence while maintaining their respective stereo-configurations to generate surugamides B–E [65]. The absolute configuration of surugamide A has been revised by total synthesis—the structure actually contains D-allo-Ile in place of D-Ile [67,68]. Surugamide F (43, Table 1) is a linear decapeptide also isolated from marine *Streptomyces* sp. JAMM992 [69,70]. Surugamides have not been initially reported as antibiotics until surugamide A was reisolated from *Streptomyces* sp. RM-27–46 by another group and was reported to have significant activity against *S. aureus*, with an MIC of 10 μM [71]. The activity data for the analogues B-E are inconclusive and the antibacterial potential of surugamides should be further explored via SAR studies.

### 2.6. Sclerotides

Sclerotides A–B (44–45, Table 1) are a class of cyclic hexapeptides isolated from marine-origin fungus *Aspergillus sclerotiorum* PT06-1, with a skeleton containing two rare residues: anthranilic acid (AA) and dehydrotryptophan (∆-Trp) [72]. Sclerotides C-E (46–48, Table 1) were isolated from a fermented rice culture of a soft coral-derived fungus, *Aspergillus sclerotiorum* SCSIO 41,031 [73]. The amino acid residue sequence for sclerotides is cyclo-Thr1-Ala2-D-Phe3-D-Ser4-AA5-∆-Trp6. The double bond in the ∆-Trp of sclerotide A is Z-type, while the opposite is true for B. Due to the presence of ∆-Trp, sclerotides A and B can be interconverted via direct light-excited free radical reactions [72]. Sclerotides A and B both exhibit mild antifungal activity for *C. albicans*, with MIC values of 7.0 µM and 3.5 µM, respectively. In addition, sclerotide B exhibits cytotoxicity (HL-60 cell line, IC50, 56.1 µM) and antibacterial activity (*P. aeruginosa*, MIC, 35.3 µM) [72]. The antimicrobial activity of sclerotides C–E has not been reported.

### 2.7. Marthiapeptide A

Marthiapeptide A (49, Table 1) is a cyclic peptide found in deep-sea-derived *Marinactinospora thermotolerans* SCSIO 00652 containing three thiazoles (Thz) and one thiazoline (Tzn) [74]. The cyclic peptide skeleton of marthiapeptide A is cyclo-Ala1-Thz2-Thz3-Thz4-Tzn5-D-Phe6-Ile7, which has been confirmed by total synthesis [74,75]. Marthiapeptide A has MICs of 4, 2, 2, and 8 μg/mL for *B. subtilis*, *Bacillus thuringiensis*, *M. luteus*, and *S. aureus*, respectively, and shows no activity against Gram-negative bacteria like *E. coli* [74]. In addition, marthiapeptide A is strongly cytotoxic, with half-maximal inhibitory concentration (IC_50_) values of 0.38–0.52 μM for a set of human cancer cell lines [74].

## 3. Cyclic Depsipeptides

Cyclic depsipeptides refer to a class of cyclic peptides with at least one ester bond involved in the cyclization in the skeleton in addition to the amide bond [76,77]. This ester bond can be formed by the participation of hydroxyl groups on the side chains of amino acid residues, like Ser and threonine (Thr). Alternatively, fatty acid chains with β-OH are used to participate in the cyclization to form lipopeptides, although in this report, lipopeptides are discussed under the category of cyclic lipopeptides. The involvement of the hydroxyl group in the cyclization allows an amino group to extend from the cyclic skeleton and be used to link other structural units, such as the cinnamic acid unit or another part of the polypeptide unit. This structural feature confers more chemical diversity and biological activity to cyclic depsipeptides.

### 3.1. Atratumycins

Atratumycins A–C (50–52, Table 2) are cyclic decapeptides with 2-alkenyl cinnamoyl moiety from marine-derived *Streptomyces atratus* SCSIO ZH16 and its engineered strain *Streptomyces atratus* SCSIO ZH16 NS-80S [58,78]. Atratumycins contain a rare amino acid unit, phenylserine (β-OH-Phe), and four D-type amino acid residues. Atratumycins share an identical cyclic skeleton of cyclo-Thr1-Ser2-D-Trp3-Asn4-(2S,3R)-β-OH-Phe5-Pro6-D-Val7-Gly8-D-Leu9-D-Tyr10 [78]. Similar to atrovimycins [79], the cinnamic acid portion of atratumycins is linked to the amino group of Thr1 and distinguishes atratumycins into A, C, and B types in order of length (C10, C12, and C14). Atratumycins A–C all have anti-TB activity with MICs of 14.6 μM, 2.3 μM, and 14.8 μM against *M. tuberculosis* H37Rv, respectively [58]. Atratumycin B has the longest cinnamic acid chain and exhibits the strongest anti-TB activity, comparable to that of atrovimycin, and both have the same cinnamic acid chain length [58]. Therefore, cinnamic acid chain length is an important factor affecting the anti-TB activity of atratumycins.

### 3.2. Nyuzenamides

Nyuzenamides A–B (53–54, Table 2) and nyuzenamide C (55, Table 2) were identified in marine suspensions of *Streptomyces* sp. N11–34 and river sediment samples of *Streptomyces* sp. DM14, respectively [80,81]. Nyuzenamides are structurally unique bicyclic decapeptides with a skeleton consisting of six general amino acid residues, Val, Leu, Asn, Thr, Gly, and proline (Pro), and four non-protein amino acid units, 3, β-dihydroxytyrosine, 4-hydroxyphenylglycine, β-hydroxyphenylalanine and hydroxyglycine, as well as a multivariate modified cinnamoyl unit attached to the Thr amine [80,81]. The propyl group of the cinnamic acid part in nyuzenamide C is epoxidized, and the double bond of the propyl group in nyuzenamides A–B is oxidatively reduced to two conformationally opposite o-hydroxy groups, where the α-position hydroxyl group of nyuzenamide B forms the methyl ether, and the β-position hydroxyl group of nyuzenamide A and the double bond at the β-position of the carbonyl group form a six-membered ring via Michael addition [80,81]. Nyuzenamides A and B do not exhibit inhibitory activity against Gram-negative and Gram-positive bacteria and yeast, but have inhibitory effects against filamentous fungi [81]. For instance, nyuzenamide A has an MIC of 3.1 μg/mL against the plant pathogen *Glomerella cingulate* and 6.3 μg/mL against the human pathogen *Trichophyton rubrum* [81]. The nyuzenamide B activity is significantly weaker than that of A. Nyuzenamide C does not show any antibacterial or antifungal activity [80]. This suggests that cyclization on the side chain of cinnamic acid has an important effect on the antibacterial activity of nyuzenamides.

### 3.3. Marformycins

Marformycins A–F (56–61, Table 2) are a class of novel cyclic heptapeptides isolated from deep-sea-derived *Streptomyces drozdowiczii* SCSIO 10,141 and characterized by a unique N-terminal formylated side chain and five non-protein amino acid residues, including piperazine acid (Pip) [82]. The sequence of amino acid residues for marformycins is cyclo-Leu1-Pip/γ-OH-Pip2-D-allo-Ile/D-Val3-O-methyl-D-Tyr4-Thr5-N-methyl-Val6, with the hydroxyl group of Thr5 involved in cyclization and the amino group attached to N-terminally formylated allo-Ile/Val7 [82]. The chemical diversity of marformycins arises from the variation in amino acid residues at positions 2, 3, and 7. Marformycins A–E exhibit very significant narrow-spectrum antibacterial activity against *M. luteus*, with MICs of 0.25, 4.0, 0.25, 0.063, and 4.0 μg/mL [82], respectively, suggesting an important role for the hydroxyl group at the γ-position of Pip and that the substitution of Val by Ile at positions 3 and 7 can enhance this activity. No marformycins exhibit cytotoxicity.

### 3.4. Ohmyungsamycins

Ohmyungsamycins A–B (62–63, Table 2) are new cyclic dodecapeptides isolated from *Streptomyces* sp. SNJ042, a strain collected from a sand beach [83]. The cyclic skeleton of ohmyungsamycins consists of 10 amino acid residues in the following order: cyclo-Val1-β-OH-Phe2-Val3-N-Me-4-OMe-Trp4-N-Val5-Val6-N-Me-Leu7-Val8-N-Me-Thr9-Thr10, where Thr10 participates in cyclization with a hydroxyl group and the amino group extends Val11/allo-Ile11-N-Me-Val12 [83]. The difference between ohmyungsamycins A and B was incorrectly attributed to the number of methyl groups in the N-terminus of Val12 in the initial report, which was later corrected to the substitution of Val11 for allo-Ile11 by performing total synthesis [84]. Ohmyungsamycin A shows significant inhibition against *B. subtilis* (MIC, 4.28 μM), *Kocuria rhizophila* (MIC, 1.07 μM), and *Proteus hauseri* (MIC, 2.14 μM) [83]. The activity of A is eight-fold that of B, indicating that allo-Ile11 has a positive effect on antibacterial activity.

There are no experimental studies demonstrating the antimicrobial mechanism of ohmyungsamycins. However, ohmyungsamycins have a highly structurally similar natural analogue, ecumicin [85], whose antimicrobial mechanism is known. Ecumicin stimulates the ATPase activity of ClpC1 and uncouples ClpC1 from the ClpP1P2 protease, causing it to lose its protein hydrolysis ability and ultimately leading to the death of *M. tuberculosis* due to disrupted protein metabolism [86,87]. Ohmyungsamycins have a high probability of targeting the same site, but this has not been confirmed by experiments. Interestingly, ohmyungsamycins have been reported to enhance host resistance to *M. tuberculosis* by activating AMPK (AMP-activated protein kinase)-dependent antibacterial autophagy and suppressing the inflammatory response caused by infection [88].

## 4. Cyclic Lipopeptides

Cyclic lipopeptides refer to a class of cyclic depsipeptides or cyclic oligopeptides modified by the presence of hydrophobic fatty acid chains in the skeleton [89,90]. Fatty acid chains are generally linked to the side chains of amino acid residues of the cyclic skeleton via amide bonds or are directly involved in cyclization through hydroxyl or amino groups on the fatty acid chain. The hydrophobicity of fatty acid chains often affects the antibacterial activity and mechanism of action of compounds, such as that of polymyxin B, daptomycin, and other cyclic lipopeptides targeting bacterial cell membranes.

### 4.1. Taromycins

Taromycins A and B (64–65, Table 3) are cyclic tridecapeptides isolated from marine actinomycete *Saccharomonospora* sp. CNQ-490 [91,92]. They are analogues of daptomycin and contain four uncommon amino acid residues: Orn, Me-glutamate (Glu), 6-chloro-trypophan (6-Cl-Trp), and 4 chloro-kynurenine (4-Cl-Kyn) [91,92]. The polypeptide skeleton of taromycins is 6-Cl-Trp1-Asn2-Aspartic acid (Asp)3-cyclo-[Thr4-Gly5-Orn6-Asp7-Ala8-Asp9-Gly10-Ala11-Me-Glu12-4-Cl-Kyn13], where the N-terminal of 6-Cl-Trp1 is attached to octa-2,4-dienoic acid or 6-methylocta-2,4-dienoic acid [92]. Taromycins and daptomycin differ in their chlorination of Trp1 and Kyn13, replacement of Ser11 by Ala11, and the length and unsaturation of the fatty acid chain. Taromycins exhibit calcium ion-dependent broad-spectrum antibacterial activity against Gram-positive bacteria, especially against several types of MRSA and *E. faecium* [91]. The activity is comparable between taromycins A and B, suggesting that divergence at the end of the fatty acid chain does not affect the antibacterial activity.

### 4.2. Hassallidins and Balticidins

Hassallidins A and B (66–67, Table 3) are cyclic lipopeptides isolated from a strain of a *Hassallia* sp. with different forms of glycosylation modifications and structures containing the rare amino acid residues dehydroaminobutyric acid (Dhb) and fatty acid chain α, β-dihydroxytetradecanoic acid (Dht) [93,94]. The cyclic peptide skeleton of hassallidin A is Dht1-Thr2-cyclo-[Thr3-Thr4-D-Tyr5-Dhb6-Gln7-Gly8-Me-Thr9-Gln10]. The hydroxyl group at Me-Thr9 is modified by mannose and β-OH of Dht1 is modified by additional rhamnose at hassallidin B [93,94]. Hassallidins have significant antifungal activity. The MIC for hassallidin A is 4–8 μg/mL against *A. fumigatus* and several strains of *Candida* species. It is twice as active as hassallidin B [93,94]. This suggests that glycosylation of the hydroxyl group at the β-position on the fat chain is perhaps unimportant.

Balticidins A-D (68–71, Table 3) are hassallidin-like lipopeptides isolated from the Baltic Sea *Cyanobacterium Anabaena cylindrica* Bio33 with antifungal activity [95]. The basic skeleton of balticidin D (69, Table 3) is dihydroxy aliphatic acid derivative (DhA)1-Thr2-cyclo-[Thr3-Thr4-D-4-β-OH-Tyr5-Dhb6-Gln7-Gly8-N-Me-Thr9-D-Gln10], in which the hydroxyl group of N-MeThr9 is linked to mannose, and the β-OH of DhA1 is linked to the disaccharide unit of arabinose and galacturonic acid [95]. Balticidin D and B (68, Table 3) are distinguished by the substitution of a Cl atom on C-13 on DhA1. Balticidin A (70, Table 3) and C (71, Table 3) are the products of ester bond breakage between Thr3 and Gln10 for B and D, respectively [95]. The compounds showed great inhibitory effect against fungi such as *Candida albicans*, *Candida krusei*, *Candida maltosa*, *Aspergillus fumigatus*, *Microsporum gypseum*, *Mucor* sp., and *Microsporum canis* [95]. However, the exact MIC values were not determined.

### 4.3. Iturins

Iturins F1, F2, and A9 (72–74, Table 3) are recently discovered new members of the classical cyclic lipopeptide iturin family isolated from *Bacillus* sp. KCB14S006 [96]. Iturin F1 (72, Table 3) consists of Tyr, Gln, 4-OH-Pro, Ser, 3 Asn, and a β-amino fatty acid chain. Iturin F1 differs from F2 (73, Table 3) in the position of the methyl group at the end of the fatty acid chain and from A9 (74, Table 3) in the replacement of 4-OH-Pro by Pro [96]. All iturins show antifungal activity, such as that against *A. flavus*, *Neurospora crassa*, *C. albicans*, *Penicillium griseofulvum*, and *Candida tropicalis* [96]. Iturins F1 and F2 show activity that is double that of A9, suggesting that Pro hydroxylation weakens the antibacterial effect.

### 4.4. Bacilotetrins

Bacilotetrins A–E (75–79, Table 3) are a class of cyclic lipid tetrapeptides isolated from marine-derived *Bacillus subtilis* 109GGC020 with three hydrophobic amino acids and one β-hydroxy fatty acid (HFA) cyclization [97,98]. The cyclic polypeptide skeleton of bacilotetrins is cyclo-Leu1-D/L-Leu2-Leu3-Dlu4-HFA5. The difference between all bacilotetrins is in the length or terminal bifurcation of the aliphatic chain. Bacilotetrins A and B show apparent antibacterial activity (8–32 μg/mL) against clinically isolated MRSA [97]. In addition, bacilotetrins C–E were reported to have inhibitory activity against *Mycoplasma hyorhinis* [98].

### 4.5. Sclerotiotides

Sclerotiotides (80–85, Table 3) are a series of cyclic tripeptides isolated from *Aspergillus sclerotiorum* PT06-1 and *Aspergillus insulicola* HDN151418 with a core skeleton consisting of N-Me-Ala, N-Me-Val, and Orn, as well as amino groups acylated by unsaturated fatty acids [99,100]. Although sclerotiotides have been reported for the 15 members A–O, most of them are inactive artifacts resulting from the isolation process. Sclerotiotides A, B, and F (80–82, Table 3) were demonstrated to have activity against *Candida albicans*, with MICs of 7.5, 3.8, and 30 µM, respectively [99]. The SAR between these indicated that the methyl group of N-Me-Val and the length of the aliphatic chain have less effect on the activity, while oxidation at the end of the aliphatic chain results in a rapid decrease in activity. Sclerotiotides M and N (78–79, Table 3) were reported to have broad-spectrum antimicrobial activity, with MICs varying from 1.56 to 25.0 µM for *B. cereus*, *B. subtilis*, *Vibrio parahemolyticus*, *Edwardsiella tarda*, *Proteus* sp., *Mycobacterium phlei*, methicillin-resistant coagulase-negative *Staphylococcus* (MRCNS), and MRSA [100].

## 5. Conclusions and Perspectives

This review described structurally well-defined peptides from marine microorganisms with antimicrobial activity reported in the last fifteen years and classified them based on structural differences into cyclic oligopeptides, cyclic depsipeptides and cyclic lipopeptides. In total, we have compiled 16 families of cyclic peptide antibiotics containing 85 compounds. Compounds of the same family, sometimes exhibit large differences in antimicrobial activity because of subtle structural differences and sometimes have no effect, which provides us with some interesting SAR information. For example, the length of the fatty chains of atratumycins significantly affects the antibacterial activity of the compounds against *M. tuberculosis*, with longer fatty chains resulting in more significant antibacterial activity [58]. The instance of taromycins, on the other hand, shows that bifurcation at the end of the fatty chain tends not to affect the strength of the antibacterial activity [92]. Differences in activity among ohmyungsamycins, meanwhile, suggest that the type of key amino acid residues in the skeleton can also have a significant effect on antibacterial activity [83]. Marformycins revealed that functional groups on the side chains of amino acid residues may also be a key factor influencing antimicrobial activity [82]. In addition, asperversiamides [55,56], atratumycins [58], and nyuzenamides [81] collectively suggest that the cinnamic acid unit in the structure may be the key to producing antibacterial activity against *M. tuberculosis*. On the other side, the abundant and diverse structures also yield different antimicrobial profiles. For example, ilamycins have potent and selective inhibition of *M. tuberculosis* [44,45], marformycins selectively and strongly inhibit *M. luteus* [82], marthiapeptide A has broad-spectrum anti-Gram-positive bacterial activity [74], and iturins [96] and sclerotides [72] exhibit impressive antifungal activity.

Regrettably, except for ilamycins and ohmyungsamycins, there is a lack of further mechanistic studies on all the other compounds, though many of them have promising antimicrobial activity. The mechanism of action and drug potential of these valuable antimicrobial skeletons are yet to be investigated in greater depth. Additionally, we note that the number of reports of polypeptide antibiotics derived from marine microorganisms is much lower than that of soil microorganisms, and reports of those capable of inhibiting Gram-negative bacteria are extremely rare. Marine microorganisms are a tremendous treasure trove for exploring active secondary metabolites, and we should pay more attention to them to further explore novel cyclic peptide antimicrobial compounds by utilizing cutting-edge analytical techniques, such as metabolomics, bioinformatics analyses, and genome mining.

## Figures and Tables

**Table 1:** Cyclic oligopeptides derived from marine microorganisms (1–49).

Ilamycins B1-G															
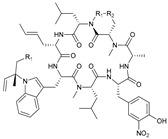					6	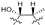		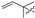	NO_2_	14		H	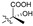	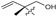	NO_2_
					7	H	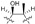	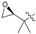	NO_2_	15		H	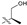	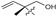	NO_2_
					8	H	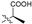	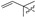	NO_2_	16		H	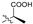	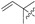	NHCHO
	R_1_	R_2_	R_3_	R_4_											
1	H	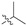	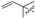	NO_2_	9	H	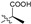	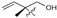	NO_2_	17		H	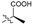	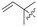	H
2	H	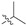	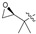	NO_2_	10	H	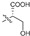	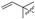	NO_2_	18		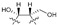		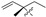	NO_2_
3	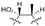		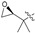	NO_2_	11	H	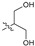	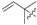	NO_2_	19		H	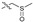	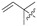	NO_2_
4	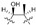		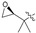	NO_2_	12	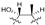		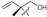	NO_2_	20		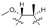		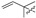	NO_2_
5	H	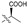	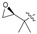	NO_2_	13	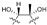		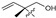	NO_2_						
Asperversiamides A–C/Asperheptatides A–D and cinnamic acid derivatives asperversiamide A															
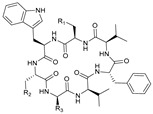					25	R_1_ = H, R_2_ = H, R_3_ = CH_3_				26	R_1_ = OH, R_2_ = CH_2_SCH_3_, R_3_ = Bn				
					27	R_1_ = OH, R_2_ = H, R_3_ = H									
					28	R_1_ = 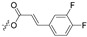 , R_2_ = 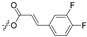 , R_3_ = CH_3_									
					29	R_1_ = 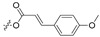 , R_2_ = H, R_3_ = CH_3_									
21	R_1_ = OH, R_2_ = OH, R_3_ = CH_3_				30	R_1_ = 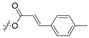 , R_2_ = 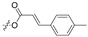 , R_3_ = CH_3_									
22	R_1_ = OH, R_2_ = H, R_3_= CH_3_														
23	R_1_ = H, R_2_ = OH, R_3_ = CH_3_				31	R_1_ = 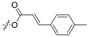 , R_2_ = 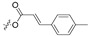 , R_3_ = CH_3_									
24	R_1_ = OH, R_2_ = OH, R_3_ = H														
Thermoactinoamide A						Desotamides A–D and G									
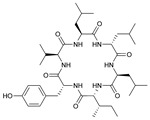						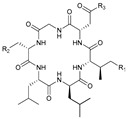				33	R_1_ = Me, R_2_ = 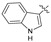 , R_3_ = NH_2_				

										34	R_1_ = H, R_2_ = 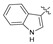 , R_3_ = NH_2_				

										35	R_1_ = Me, R_2_ = 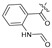 , R_3_ = NH_2_				
					37	R_1_ = Me, R_2_ = 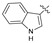 , R_3_ = OH									
32															
										36	R_1_ = Me, R_2_ = 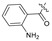 , R_3_ = NH_2_				
Surugamides A–E					Surugamide F			Sclerotides A–B and D					Sclerotide C		
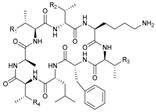					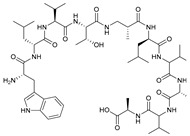			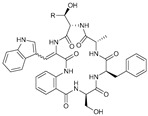					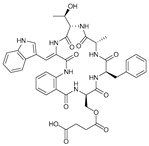		
								44	R = Me, △ = *Z*						
38	R_1_ = CH_3_, R_2_ = CH_3_, R_3_ = CH_3_, R4 = CH_3_					43		45	R = Me, △ = *E*				46		
39	R_1_ = CH_3_, R_2_ = H, R_3_ = CH_3_, R4 = CH_3_							47	R = H, △ = *Z*						
40	R_1_ = CH_3_, R_2_ = CH_3_, R_3_ = H, R4 = CH_3_					Marthiapeptide A							Sclerotide E		
41	R_1_ = H, R_2_ = CH_3_, R_3_ = CH_3_, R4 = CH_3_					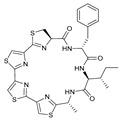							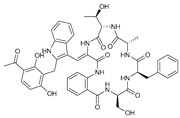		
42	R_1_ = CH_3_, R_2_ = CH_3_, R_3_ = CH_3_, R4 = H														

						49							48		

**Table 2:** Cyclic depsipeptides derived from marine microorganisms (50–63).

Atratumycins A–C				Nyuzenamides A–C				
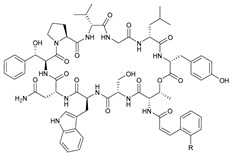				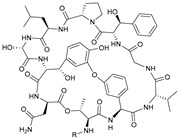		53	R = 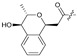	
						54	R = 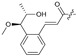	
						55	R = 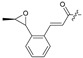	
50	R = 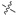	51	R = 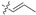					
		52	R = 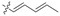					
Marformycins A–F						Ohmyungsamycins A–B		
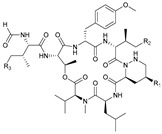			56	R_1_ = H, R_2_ = CH_3_, R_3_ = H		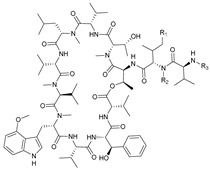		
			57	R_1_ = H, R_2_ = CH_3_, R_3_ = CH_3_				
			58	R_1_ = OH, R_2_ = H, R_3_ = CH_3_				
			59	R_1_ = OH, R_2_ = CH_3_, R_3_ = CH_3_				
			60	R_1_ = OH, R_2_ = H, R_3_ = H				
			61	R_1_ = H, R_2_ = H, R_3_ = H				
						62	R_1_ = H, R_2_ = H, R_3_ = CH_3_	
						63	R_1_ = CH_3_, R_2_ = H, R_3_ = CH_3_	

**Table 3:** Cyclic lipopeptides derived from marine microorganisms (64–85).

Taromycins A–B						Hassallidins A–B									
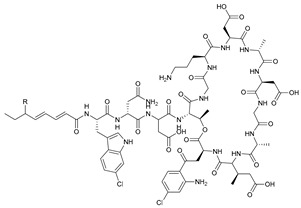						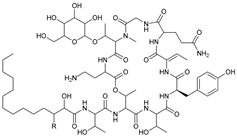					66	R = OH	67	R = 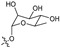	

	64	R = H	65	R = CH_3_											
Balticidins B and D						Balticidins A and C									
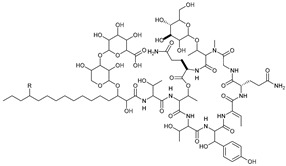						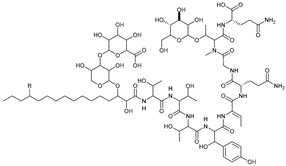									
	68	R = Cl	69	R = H			70	R = Cl	71	R = H					
Iturins F1, F2, and A9									Bacilotetrins A–E						
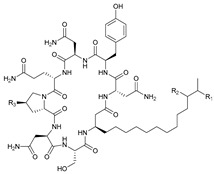				72	R_1_ = CH_3_, R_2_ = H, R_3_ = OH				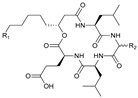						
				73	R_1_ = H, R_2_ = CH_3_, R_3_ = OH										
				74	R_1_ = H, R_2_ = CH_3_, R_3_ = H										
	
									75	R_1_ = 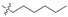 , R_2_ = 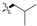					
									76	R_1_ = 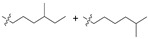 , R_2_ = 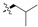					

Sclerotiotides A, B, F, M-O									77	R_1_ = 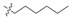 , R_2_ = 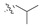					
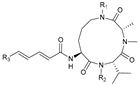				83	R_1_ = H, R_2_ = CH_3_, R_3_ = 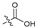										
									78	R_1_ = 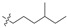 , R_2_ = 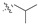					
				84	R_1_ = H, R_2_ = CH_3_, R_3_ = 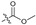										
									79	R_1_ = 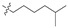 , R_2_ = 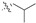					
80	R_1_ = H, R_2_ = H, R_3_ = 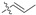			85	R_1_ = CH_3_, R_2_ = CH_3_, R_3_ = 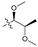										
81	R_1_ = H, R_2_ = CH_3_, R_3_ = CH_3_														
82	R_1_ = H, R_2_ = CH_3_, R_3_ = CHO														

## Data Availability

Not applicable.
